# Population-based bloodstream infection surveillance in rural Thailand, 2007–2014

**DOI:** 10.1186/s12889-019-6775-4

**Published:** 2019-05-10

**Authors:** Julia Rhodes, Possawat Jorakate, Sirirat Makprasert, Ornuma Sangwichian, Anek Kaewpan, Thantapat Akarachotpong, Prasong Srisaengchai, Somsak Thamthitiwat, Supphachoke Khemla, Somkid Yuenprakhon, Wantana Paveenkittiporn, Anusak Kerdsin, Toni Whistler, Henry C. Baggett, Christopher J. Gregory

**Affiliations:** 1 Global Disease Detection Center, Thailand Ministry of Public Health (MOPH) – United States Centers for Disease Control and Prevention (CDC) Collaboration, Nonthaburi, Thailand; 2 Division of Global Health Protection, Center for Global Health, CDC, Atlanta, GA USA; 3 Nakhon Phanom General Hospital, Nakhon Phanom, Thailand; 4 Sa Kaeo Crown Prince Hospital, Sa Kaeo, Thailand; 5 Department of Medical Sciences, National Institute of Health, Ministry of Public Health, Nonthaburi, Thailand; 6 Faculty of Public Health, Kasetsart University Chalermphrakiat, Sakon Nakhon Province, Thailand

**Keywords:** Bloodstream infections, Community-acquired infections, Healthcare-associated infections, Antimicrobial resistance, Population-based surveillance, Thailand, Global health security

## Abstract

**Background:**

Bloodstream infection (BSI) surveillance is essential to characterize the public health threat of bacteremia. We summarize BSI epidemiology in rural Thailand over an eight year period.

**Methods:**

Population-based surveillance captured clinically indicated blood cultures and associated antimicrobial susceptibility results performed in all 20 hospitals in Nakhon Phanom (NP) and Sa Kaeo (SK) provinces. BSIs were classified as community-onset (CO) when positive cultures were obtained ≤2 days after hospital admission and hospital-onset (HO) thereafter. Hospitalization denominator data were available for incidence estimates for 2009–2014.

**Results:**

From 2007 to 2014 a total of 11,166 BSIs were identified from 134,441 blood cultures. Annual CO BSI incidence ranged between 89.2 and 123.5 cases per 100,000 persons in SK and NP until 2011. Afterwards, CO incidence remained stable in SK and increased in NP, reaching 155.7 in 2013. Increases in CO BSI incidence over time were limited to persons aged ≥50 years. Ten pathogens, in rank order, accounted for > 65% of CO BSIs in both provinces, all age-groups, and all years: *Escherichia coli*, *Klebsiella pneumoniae*, *Burkholderia pseudomallei*, *Staphylococcus aureus*, *Salmonella* non-typhi spp., *Streptococcus pneumoniae*, *Acinetobacter* spp., *Streptococcus agalactiae, Streptococcus pyogenes, Pseudomonas aeruginosa*. HO BSI incidence increased in NP from 0.58 cases per 1000 hospitalizations in 2009 to 0.91 in 2014, but were higher (ranging from 1.9 to 2.3) in SK throughout the study period. Extended-spectrum beta-lactamase production among *E. coli* isolates and multi-drug resistance among *Acinetobacter* spp. isolates was common (> 25% of isolates), especially among HO cases (> 50% of isolates), and became more common over time, while methicillin-resistance among *S. aureus isolates* (10%) showed no clear trend. Carbapenem-resistant *Enterobacteriaceae* were documented in 2011–2014.

**Conclusions:**

Population-based surveillance documented CO BSI incidence estimates higher than previously reported from Thailand and the region, with temporal increases seen in older populations. The most commonly observed pathogens including resistance profiles were similar to leading pathogens and resistance profiles worldwide, thus; prevention strategies with demonstrated success elsewhere may prove effective in Thailand.

**Electronic supplementary material:**

The online version of this article (10.1186/s12889-019-6775-4) contains supplementary material, which is available to authorized users.

## Background

Bloodstream infections (BSI) are important causes of morbidity and mortality with incidence rates comparable to stroke, acute myocardial infarction and trauma [1] but the public health threat of BSI has been poorly characterized in Southeast Asia. BSI burden estimates are often limited to North America and Europe due to a lack of population-based studies in other parts of the world [2]. Population-based BSI incidence estimates from Southeast Asia are especially scarce, but pathogen-specific studies and reports of antimicrobial resistant pathogens indicate a substantial BSI burden in Southeast Asia [3–7]. A systematic review of community-acquired BSIs in south and Southeast Asia, found that 9% of hospitalized, febrile patients with blood cultures collected had bacteremia and 9% of these patients died in-hospital [8].

While this information is useful, additional descriptions of BSI epidemiology from South East Asia are needed to characterize the public health threat by: 1) allowing for comparison between BSI disease burden and trends versus other health conditions, 2) assessing the relative importance of specific BSI pathogens, and 3) identifying high-risk sub-populations for development of effective treatment regimens and targeting of public health prevention interventions. Estimation of the overall BSI burden is particularly useful as prevention strategies are not necessarily pathogen specific and overall estimates are needed to evaluate the potential impact of infection control and antimicrobial resistance programs. Furthermore, characterization of BSI epidemiology facilitates rapid detection and containment of public health threats at their source, thereby enhancing global health security.

From 2005 through 2014, the Thailand Ministry of Public Health - US Centers for Disease Control and Prevention (CDC) Collaboration, together with the Nakhon Phanom and Sa Kaeo Provincial Health Offices, conducted population-based BSI surveillance in Sa Kaeo and Nakhon Phanom provinces. In this paper we summarize the overall findings.

## Methods

### Setting

Sa Kaeo is located in eastern Thailand near the Cambodian border; Nakhon Phanom is in northeast Thailand near the Laos border. These two rural provinces have a combined population of 1.1 million. All 20 hospitals in the two provinces participated, including two provincial hospitals (225–327 bed referral centers) and 18 peripheral, district hospitals (10–140 beds). There were no private hospitals in either province and Thai citizens are provided with health care at minimal or no cost.

### Specimen collection and processing

Blood cultures were performed as clinically indicated per physician request among hospitalized patients. From 2007 to 2010, we encouraged blood cultures for hospitalized patients with suspected pneumonia and for patients aged ≤5 years old with possible sepsis, by reimbursing hospitals for the costs of culture for these patients. Nurses, regularly trained in phlebotomy, collected specimens from a single peripheral site. According to protocol targets, specimens from district hospitals were transported at 15–30 °C within 24 h of collection to provincial hospital laboratories, where all specimens were processed using an automated blood culture system (BacT/ALERT® 3D, bioMérieux, U.S.A.). Nurses completed a form with patient details including antibiotic use in the last 72 h.

Each blood specimen was divided into a bottle optimized for aerobic growth [BacT/ALERT FAN Aerobic (FA) for ages ≥ 5 years (target volume of 10 mL) and BacT/ALERT Pediatric FAN (PF) for ages < 5 years (target volume of 4 mL)] and a bottle for enhanced growth of mycobacteria, fungal pathogens, and other fastidious agents [BacT/ALERT Mycobacteria Blood (MB) (target volume of 3 mL)]. If blood volume was insufficient to inoculate both bottles at targeted levels, the FA/PF bottle was prioritized. The protocol was modified in October 2011; thereafter, MB bottles were not routinely processed, but available by physician request, (e.g. for cases of suspected tuberculosis or *Burkholderia pseudomallei* infection) [9]. FA/PF and MB bottles were incubated in the BacT/ALERT system at 35 °C for up to 5 and 42 days respectively, or until the instrument signaled a positive result for growth (i.e., alarm positive). Media from alarm positive bottles was sub-cultured onto sheep blood, chocolate and MacConkey agar plates and incubated overnight at 35 °C. Standard biochemical testing was used for identification [10]. From 2012, API® identification strips (bioMérieux, U.S.A.) were used when standard testing was not definitive. Confirmatory identification of all pathogens was performed at Thailand’s National Institute of Health through the end of 2010, when confirmatory testing was limited to selected species (e.g. *Streptococcus* spp*., B. pseudomallei, Salmonella* non-typhi spp*., Vibrio* spp. and unusual pathogens). Confirmatory testing was discontinued in 2012; however participation in national External Quality Assurance (EQA) programs has been ongoing throughout the surveillance period and an international EQA program (Royal College of Pathologists of Australia Quality Assurance Programs Pty. Ltd., Australia) was initiated in 2011 and continued throughout the remainder of the surveillance period.

Antimicrobial susceptibility testing for 1) extended spectrum beta-lactamase (ESBL) *E. coli* and *K. pneumoniae,* 2) methicillin-resistant *S. aureus* (MRSA), 3) vancomycin-resistant S. aureus (VRSA), 4) penicillin-resistant *S. pneumoniae*, and 5) carbapenem-resistant enterobacteriaceae (CRE) was performed according to the Clinical and Laboratory Standards Institute guidelines [11]. ESBL screening test results were used to determine ESBL status when ESBL *E. coli* and *K. pneumoniae* confirmatory testing was not available. This definition was based on prior work which found screening test sensitivity > 95%, specificity > 90%, and > 90% agreement with confirmatory tests for both *E. coli* and *K. pneumoniae* [12, 13]. All CRE cases were confirmed by retesting at the Thailand NIH. *Acinetobacter* spp. isolates were defined as multi-drug resistant (MDR) if they were resistant to 3 or more of the following drug classes by disc diffusion testing: aminoglycosides (Amikacin or Gentamycin); cephalosporins (Cefotaxime or Ceftazidime or Cefoperazone); fluoroquinolones (Ciprofloxacin); carbapenems (Imipenem or Meropenem) [14].

Serotyping for *S. pneumoniae* was performed using multiplex PCR [15]. For isolates that could not be typed by this method, Quellung serotyping was done at the Streptococcus Reference Laboratory, U.S. CDC in Atlanta, Georgia. Serotyping for *Haemophilus influenza* was performed using real-time, multiplex PCR [16] at the Thailand National Institute of Health.

### Definitions and population data

**Fig. 1:**
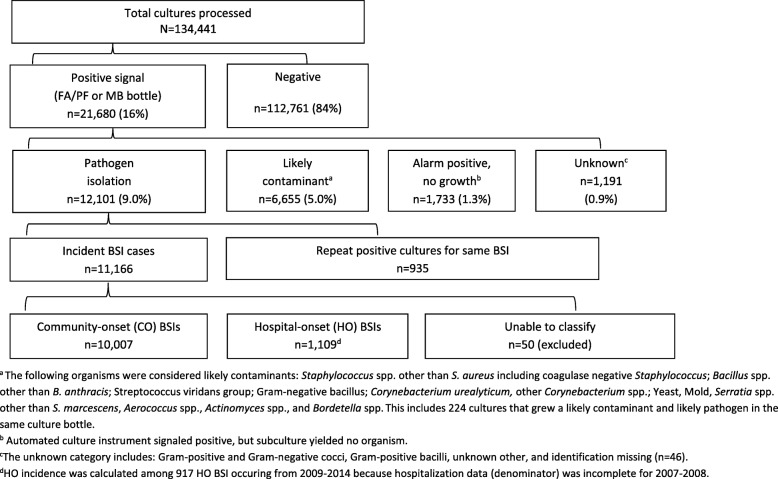
Flow diagram of blood cultures results, Sa Kaeo and Nakhon Phanom provinces, Thailand, 2007–2014

Bacterial isolates were defined as likely pathogens if at least one culture bottle (FA/PF or MB) grew an organism likely responsible for infection [17] and did not grow a likely contaminant in the same bottle; *S. pneumoniae*, *B. pseudomallei* and *Salmonella* non-typhi spp. were considered pathogens regardless of other isolate growth. Common skin and environmental organisms were considered likely contaminants (Fig. 1). For patients with multiple positive cultures, the first positive culture to grow a pathogen was included as a BSI case. Repeat positive cultures that grew the same species within 30 days were excluded with the exception of *B. pseudomallei* for which cases were only counted once regardless of the timing of subsequent positive cultures.

BSI cases were classified as hospital-onset (HO) or community-onset (CO) based on definitions adapted from MRSA surveillance [18]; HO cases were defined as those with pathogens from blood specimens collected > 2 days after the hospital admission date and CO cases were those collected ≤2 calendar days after admission.

CO incidence rate denominators were derived from Sa Kaeo and Nakhon Phanom provincial population projections for 2010–2014 from the 2010 National Economic and Social Development Board (NESDB) of Thailand [19]. For the period 2007–2009, age-stratified official intercensal estimates were not available. Instead, NESDB provided revised overall provincial population estimates for 2007–2009 based on the 2010 census. To derive age-specific population estimates we applied the 2010 NESDB age distribution to 2007–2009 NESDB overall provincial population estimates [20].

HO incidence rate denominators were calculated using the number of hospitalizations from 2009 to 2014 as found in hospital administrative databases (Naorat, S., Piralam, B., personal communication, 2015). In Sa Kaeo, four district hospitals were missing 2009 data. Together, these hospitals account for ≤15% of annual hospitalizations in our surveillance system. After careful examination of hospitalization trends, we imputed 2009 hospitalization data with observed 2010 data for these four hospitals. Military hospitals in Sa Kaeo and Nakhon Phanom provinces were also excluded from HO incidence calculations, as hospitalization data was not available for Nakhon Phanom and only available for two years in Sa Kaeo (2013–2014). Military hospitals comprised < 1% of all hospitalizations in each year with available data.

Annual incidence estimates were calculated as the number of cases in each year divided by the population estimate for each year and reported as cases per 100,000 persons. Ninety-five percent confidence intervals (CIs) on incidence estimates were calculated based on a Poisson distribution using the exact method. The statistical significance of trends over time in incidence rates was calculated by fitting a linear regression model with ‘year’ as the only predictor of annual incidence estimates. *P*-values <.05 for the year variable coefficient indicated significant trends over time. Analyses were conducted using SAS version 9.3 (SAS Institute Inc., Cary, NC, U.S.A.)

## Results

From 2007 to 2014 a total of 134,441 blood cultures were processed for 128,503 patients. The annual number of patients cultured increased from 15,727 in 2007 to 17,893 in 2014. Blood volume targets were met for FA/PF bottles for 93% of cultures among persons ≥5 years old and 28% of children < 5 years old. District hospitals accounted for 45% of blood cultures and 44% of pathogen positive cases, with the remainder coming from provincial hospitals. Specimens from district hospitals were placed in the BacT/ALERT instrument within 24 h of collection for 89% of cultures. Overall, 16% of processed cultures signaled positive: a pathogen was isolated from 9.0%, 5.0% grew a likely contaminant only, 1.3% were alarm positive, but had no growth on sub-culture and the remainder were not identified (Fig. 1). Cultures positive for only contaminants ranged from 4.1% in 2014 to 6.1% in 2012.

Of 11,166 BSIs, 10,007 were CO, 1109 were HO, and 50 could not be determined as CO vs HO (Fig. 1). In provincial hospitals, equal proportions of CO and HO cases came from surgical wards (16%) and from non-surgical, in-patient wards (61%); however, the remaining CO cases came from emergency rooms (16%) and intensive care units (ICUs) (6%), while all of remaining HO cases came from ICUs (24%), (ward data available for provincial hospitals from 2010 to 2014 only).

From 2007 to 2014, overall CO BSI incidence was 110 (95% CI: 98, 123) cases per 100,000 population with a significant increase over time (Table 1). Overall HO BSI incidence was 1.3 (95% CI: 0.9, 1.7) cases per 1000 hospitalizations with no significant trend. CO BSI incidence was highest for persons 65+ years, followed by those age 50–64 years, and children < 5 years old. HO BSI incidence was also highest among older persons but did not differ between 50–64 year olds and 65+ year olds. Increases in CO BSI incidence over time were limited to persons aged 50 years and older. Increases in incidence, together with increasing populations in older age groups, translated into increases in case counts: from 263 cases in 2007 to 492 cases in 2014 among 50–64 year olds (46% increase); from 343 cases in 2007 to 572 cases in 2014 among those 65 years and older (40% increase).

**Table 1:** Annual community-onset (CO) and hospital-onset (HO)^a^ BSI incidence^b,c^ by age in Sa Kaeo and Nakhon Phanom provinces Thailand, 2007-2014

Age (yrs.)	Annual BSI Incidence (95% CI)								p-value for trend	Overall incidence (95% CI)
	2007	2008	2009	2010	2011	2012	2013	2014		
Overall										
CO (N=10,007)	91 (80, 103)	91 (79, 102)	111 (99, 123)	118 (105, 131)	100 (88, 111)	108 (96, 120)	133 (119, 146)	130 (117, 143)	<.05	110 (98, 123)
HO (N=917)			1.1 (0.71, 1.5)	1.2 (0.8, 1.6)	1.4 (0.9, 1.8)	1.5 (1.0, 1.9)	1.1 (0.8, 1.5)	1.5 (1.0, 1.9)	NS	1.3 (0.9, 1.7)
<5										
CO (N=522)	107 (86, 115)	82 (63, 90)	57 (41, 64)	77 (59, 84)	46 (32, 52)	84 (65, 90)	71 (53, 76)	71 (53, 76)	NS	75 (57, 93)
HO (N=107)			0.6 (0.3, 0.9)	0.7 (0.3, 1.0)	0.8 (0.5, 1.2)	0.9 (0.5, 1.2)	0.6 (0.3, 0.9)	0.7 (0.4, 1.1)	NS	0.7 (0.4, 1.0)
5-14										
CO (N=187)	15 (10, 42)	15 (9.6, 41)	9.5 (5.2, 28)	13 (7.7, 35)	10 (5.7, 29)	7.2 (3.5, 21)	13 (7.6, 34)	13 (7.7, 34)	NS	12 (7.1, 17)
HO^d^ (N=11)				NA	NA	NA	NA	NA	-	0.2 (0.0, 0.5)
15-49										
CO (N=2,739)	56 (50, 62)	59 (52, 65)	72 (65, 79)	70 (63, 77)	60 (53, 66)	53 (47, 59)	66 (60, 73)	64 (57, 70)	NS	62 (56, 69)
HO (N=234)			0.9 (0.6, 1.2)	1.0 (0.7, 1.3)	1.0 (0.7, 1.3)	0.8 (0.5, 1.0)	0.7 (0.5, 1.0)	0.8 (0.5, 1.0)	NS	0.9 (0.6, 1.1)
50-64										
CO (N=3,019)	137 (120, 153)	144 (127, 161)	193 (173, 213)	203 (182, 223)	176 (157, 194)	184 (165, 203)	247 (225, 268)	225 (205, 245)	<.01	190 (171, 209)
HO (N=262)			2.1 (1.4, 2.7)	2.4 (1.7, 3.1)	1.9 (1.3, 2.5)	2.7 (2.0, 3.4)	1.7 (1.1, 2.2)	3.0 (2.2, 3.7)	NS	2.3 (1.6, 3.0)
65+										
CO (N=3,540)	342 (306, 378)	328 (292, 364)	424 (383, 464)	466 (423, 509)	385 (347, 423)	448 (407, 488)	500 (458, 541)	501 (460, 542)	<.01	426 (387, 466)
HO (N=303)			1.7 (1.2, 2.3)	1.9 (1.3, 2.5)	2.5 (1.8, 3.2)	2.8 (2.1, 3.5)	2.4 (1.8, 3.1)	2.6 (1.9, 3.2)	.06	2.3 (1.7, 3.0)

^a^Denominator data for HO incidence calculations available after 2008.

^b^CO incidence: cases/100,000 population

^c^HO incidence: cases/1,000 hospitalizations

^d^Annual incidence estimates not shown as yearly case counts were <5

**Fig. 2:**
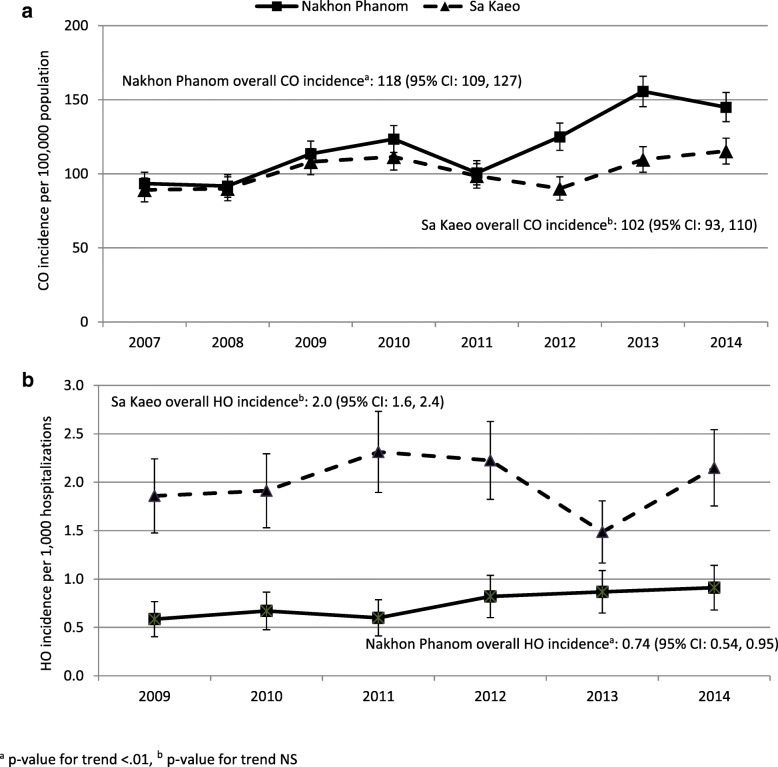
**a**/**b** Annual community-onset (CO) and hospital-onset (HO) BSI incidence in Sa Kaeo and Nakhon Phanom provinces, Thailand, 2007–2014

CO BSI incidence (95% CI) was similar in Sa Kaeo and Nakhon Phanom and ranged between 89 (81, 97) and 124 (114, 133) cases per 100,000 population until 2011 (Fig. 2a). Afterwards, incidence increased to a high of 156 (145, 166) in 2013 in Nakhon Phanom (*p*-value for trend < 0.01), while no clear increase was observed in Sa Kaeo. The higher 2013–2014 incidence in Nakhon Phanom corresponded with increases in several pathogens: *E. coli* (45% increase*)*, *K. pneumoniae* (33%), *S. aureus* (36%), *S. pneumoniae* (38%), and *Pseudomonas aeruginosa* (32%).

HO BSI incidence was higher in Sa Kaeo province compared to Nakhon Phanom (Fig. 2b). In Sa Kaeo, HO BSI incidence ranged from 1.9 to 2.3 cases per 1000 hospitalizations and did not increase significantly over time; whereas, in Nakhon Phanom HO incidence (95% CI) increased from 0.58 (0.40, 0.77) cases per 1000 hospitalizations in 2007 to 0.91 (0.68, 1.14) in 2014, *p* < 0.01. Provincial (i.e. referral) hospitals had HO BSI incidences 2.7–6.6 times higher than district level hospitals (data not shown).

Gram-negative bacteria accounted for 73% of CO BSI, Gram-positive bacteria 25%, and fungi 2.2% (1.6% *Cryptococcus neoformans* (*n* = 165); 0.6% *Candida* spp. (*n* = 56)). CO Gram-negative BSIs peaked during July to October largely due to seasonal increases in *B. pseudomallei*. There was little difference in CO Gram-positive BSI frequency throughout the year (Additional file 1: Figure S1).

**Fig. 3:**
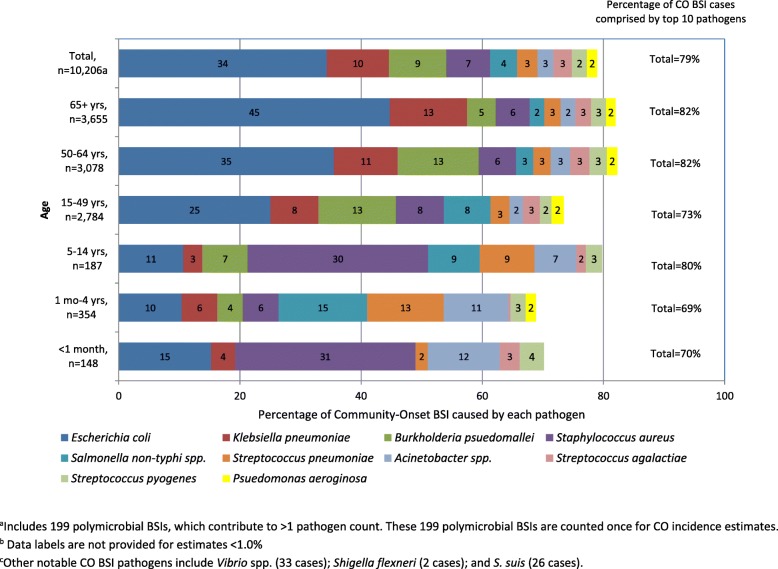
Ten most commonly observed community-onset (CO) BSI pathogens by age in Sa Kaeo and Nakhon Phanom provinces, Thailand, 2007–2014

Ten pathogens accounted for more than two-thirds of CO BSIs in both provinces, in all age-groups (except newborns < 1 month old), and in all years: *E. coli*, *K. pneumoniae*, *B. pseudomallei*, *S. aureus*, *Salmonella* non-typhi spp*.*, *S. pneumoniae*, *Acinetobacter* spp*.*, *S. agalactiae, S. pyogenes*, and *P. aeruginosa*. Polymicrobial infections were observed in 4.1% of cases with *E. coli* and *K. pneumoniae* co-infections being the most common. Pathogen distributions for patients aged 15–49, 50–64, and 65 years and older were similar with *E. coli* as the most common pathogen, followed by *B. pseudomallei* and *K. pneumoniae* (Fig. 3), though *E. coli* accounted for an increasing proportion of cases with age. *C. neoformans* accounted for 4.7% of cases among the 15–49 year olds and less than 2% among all other age groups. Pathogen distributions varied substantially among children. *S. aureus* accounted for 31% and 30% of CO BSIs among newborns and 5–14 year-olds, respectively, but only 6% among 1–4 year olds. Instead, two potentially vaccine preventable pathogens, *S. pneumoniae, and H. influenzae* were common causes of BSIs among 1–4 year olds: *S. pneumoniae* accounted for 13% and *H. influenzae* 7.6%. Serotypes included in the 13-valent pneumococcal conjugate vaccine (PCV13) accounted for 73% of all *S. pneumoniae* cases (159 of 218 with serotype available) and 92% among children < 5 years old (33 of 36 with serotype available). Vaccine preventable *H. influenzae* serotype B (Hib) accounted for 42% (17 of 40 with serotype available) of all *H. influenzae* cases and 52% among children < 5 years old. No cases of *Neisseria meningitidis* were found.

**Fig. 4:**
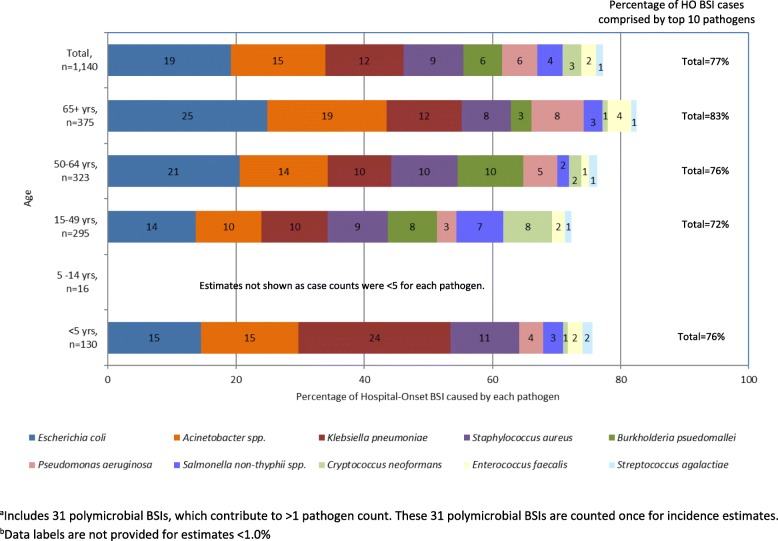
Ten most commonly observed hospital-onset (HO) BSI pathogens by age in Sa Kaeo and Nakhon Phanom provinces

*Acinetobacter* spp., *P. aeruginosa, and Enterococcus faecalis* were more common among HO cases compared to CO cases, while *E. coli*, *B. pseudomallei*, and *S. pneumoniae* were less common among HO cases than CO cases (Fig. 4). Of the *B. pseudomallei* cases that met the HO definitions, 84% (*n* = 59) represented the first blood culture performed during the hospital stay. Twenty of the fifty-nine first cultures (34%) were taken on Hospital Day 3 just missing the CO cut-point, an additional 24% (14/59) were taken on Hospital Day 4 and 86% (51/59) were taken within the first week of hospitalization.

ESBL production among *E. coli* and *K. pneumoniae* isolates*,* and MDR among *Acinetobacter* spp. isolates, was common (> 25% of isolates). CO ESBL-producing *E. coli* and MDR *Acinetobacter* spp. became more common over time. The proportions of ESBL producing *E. coli* and *K. pneumoniae*, MRSA, and MDR *Acinetobacter* were 2–3 times higher among HO cases compared to CO cases (Table 2). Neither vancomycin resistant *S. aureus* (VRSA), nor penicillin-resistant *S. pneumoniae* were observed. Eight cases of CRE were identified between 2011 and 2014; six of these cases were also ESBL-producers.

**Table 2:** Antimicrobial resistance and temporal trends among community-onset (CO) and hospital-onset (HO) BSI pathogens in Sa Kaeo and Nakhon Phanom provinces, Thailand, 2007-2014

	CO		HO	
Antimicrobial resistance issue	% Resistant^a^ (n/N tested)	Temporal Trends	% Resistant^a^ (n/N tested)	Temporal Trends
Extended-spectrum beta-lactamase (ESBL) producing *E. coli*^*b*^	27% (763/3047)	Increased from 20% in 2008-2010 to 28% in 2011-2014	51% (105/203)	No clear trend, range: 38% in 2010 to 69% in 2011
ESBL producing *K. pneumoniae*	23% (213/912)	No clear trend, range: 19% in 2014 to 30% in 2012	55% (68/123)	No clear trend, range: 23% in 2009 to 75% in 2013
Carbapenem-resistant Enterobacteriaceae (CRE)	5 cases	No cases before 2011. 5 cases total : 2011 (1), 2013 (1), 2014 (3)	3 cases	No cases before 2012. 3 cases total: 2012 (2), 2014 (1).
Methicillin-resistant *S. aureus* (MRSA)^c^	7% (55/744)	No clear trend	20% (22/111)	No clear trend
Vancomycin-resistant *S. aureus* (VRSA)^d^	0	0%	0	0%
Penicillin-resistant *S. pneumoniae*^*e*^	0	0%	0	0%
Carbapenem-resistant *Acinetobacter* spp.	34% (84/242)	Increased from 16% in 2007 to >40% in 2008 and then decreased to 23% in 2014	69% (101/146)	No clear trend, range: 91% in 2010 to 46% in 2012
MDR *Acinetobacter* spp.^f^	26% (56/219)	Increased from <10% in 2007 to >45% in 2010; no clear trend from 2011 (35%) to 2014 (23%)	70% (97/138)	Decrease of borderline significance (p<0.10) from 2008 to 2014

^a^Calculated among isolates tested

^b^Data available for 2008-2014. ESBL-producing criteria: ≥5 mm increase in the zone of growth inhibition of ceftazidime/clavulanic acid combination disc compared to ceftazidime or cefotaxime discs alone. If confirmatory testing was not available, ESBL screening test results were used: zone of inhibition for ceftazidime ≤22 mm or cefotaxime ≤27 mm

^c^MRSA criteria: prior to 2007, oxacillin disk diffusion with a zone of inhibition <10mm; from 2007, cefoxitin disk diffusion with a zone of inhibition <21 mm

^d^VRSA criteria: MRSA isolates tested for vancomycin minimum inhibitory concentration (MIC) ≤2 ug/ml as determined by E-test (bioMérieux, U.S.A.)

^e^Penicillin resistance criteria: oxacillin disc diffusion zone of inhibition <20 mm with confirmation by a penicillin MIC ≥8 μg/mL by E-test

^f^MDR *Acinetobacter* criteria: resistant to 3 or more drug classes: aminoglycosides, cephalosporins, fluoroquinolones, carbapenems

^g^CRE criteria: resistant to imipenem, meropenem, doripenem, or ertapenem by disc diffusion (zone of inhibition <20 mm)

## Discussion

Population-based surveillance capturing > 130,000 blood cultures over 8 years documented an overall CO BSI incidence of 110 cases per 100,000 population, substantially higher than previous reports from Thailand and the region. This disease incidence is comparable to that of injuries due to road traffic accidents (86 cases per 100,000 population) and all-site cancers (~ 150 cases per 100,000 population) according to WHO Global Disease Burden estimates for SE Asia [21].

Our 2007–2010 Nakhon Phanom CO BSI incidence estimates are considerably higher than estimates from 10 provincial hospitals in northeast Thailand reported by Kanoksil et al.: 91 vs 32.9 (2007); 91 vs 34.6 (2008); 111 vs 38.2 (2009); and 118 vs 31.1 (2010) [22]. Our comparatively higher estimates likely result from three methodological differences. First, the NESDB Nakhon Phanom population estimates used in our incidence calculations are smaller than the estimates used by Kanoksil et al. [23]. Differing population estimates cannot entirely account for this disparity as our estimates are still significantly higher when we use Department of Provincial Administration population estimates (data not shown). Second, we included district level hospitals. Kanoksil et al. only captured cases from provincial level hospitals with the rationale that severely ill patients are transferred to provincial hospitals, yet we found that 44% of pathogen positive cases were collected at district hospitals in Nakhon Phanom province. When our Nakhon Phanom CO BSI estimates were limited to the provincial level hospital, they ranged between 38.8 cases (2007) and 51.1 (2014), which is comparable to Kanoksil et al.’s estimate for Nakhom Phanom province only: 57.8 (2010). Third, our population-based surveillance system included laboratory capacity strengthening activities, reimbursement for cultures for suspected pneumonia cases and children < 5 years old, and on-going training for nurses and medical technologists, which may have increased frequency of blood culture collection, decreased contamination rates, and increased culture sensitivity. Previously published bacteremia incidence estimates for Sa Kaeo province are not available. Although our CO BSI incidence is higher than previously reported, we almost certainly underestimated the true burden as we only captured hospitalized patients, pre-culture antibiotic use was common which lowered culture yield [24], and blood volume targets were frequently missed for children < 5 years old.

Our observed increases in HO BSI incidence in Nakhon Phanom are consistent with previous reports from Northeast Thailand [25]. Unfortunately, our incidence estimates are not directly comparable with those from Hongsuwan et al. as we report cases per 1000 hospitalizations, while they reported cases per 1000 hospital days. We cannot fully explain the notably higher HO BSI incidence rates in Sa Kaeo province compared to Nakhon Phanom, but it may be attributable to differences in physician practices which is consistent with our observation that hospital stays are longer in Sa Kaeo province compared to Nakhon Phanom and blood cultures are more likely to be performed on patients who have been hospitalized for > 2 days in Sa Kaeo compared to Nakhon Phanom (data not shown). The increasing HO BSI incidence in Nakhon Phanom, together with high HO BSI incidence rates in Sa Kaeo province, points to a substantial, and potentially growing, HO BSI burden in rural Thailand.

With the notable exceptions of *B. pseudomallei* and *Salmonella* non-typhi spp., our most commonly identified CO pathogens, *E. coli, K. pneumoniae, S. aureus*, and *S. pneumoniae*, are well-recognized as leading causes of BSI worldwide [26] and in the Asia region [27]. The higher frequency of *B. pseudomallei* in our setting was expected as melioidosis is endemic in these areas of Thailand [6, 28], but virtually absent from the study settings included in Laupland’s review (e.g. Sweden, U.S.A., Denmark, Finland, and England). Similarly, non-typhoidal *Salmonella* spp. were uncommon in these high income countries, but a documented, common BSI pathogen in rural Thailand [22], Laos [29], and the region [8].

We observed substantial increases in CO ESBL producing *E. coli and* MDR *Acinetobacter* spp. infections and emergence of CRE. Previously, we described a MDR prevalence of 31% for healthcare-associated (HCA) *Acinetobacter* bacteremia cases compared to 24% for non-HCA cases for 2005–2008 [30]. Healthcare-associated risk factor data needed to classify CO cases HCA vs. non-HCA were not available for the current analysis; however MDR *Acinetobacter* prevalence has clearly increased over time, representing 45–50% of *Acinetobacter* bacteremia cases from 2007 to 2014.

This is a laboratory-based surveillance system, supplemented with limited clinical data, which restricts our ability to describe several important patient characteristics, including outcome. We cannot distinguish primary bloodstream infections from BSI secondary to a focal infection, and in some cases, pathogen vs. contaminant. Likewise, we could only crudely classify cases by community- and hospital-onset of infection which is not always a true reflection of the timing of symptom onset and ultimately, whether the pathogen was acquired in the community, in the hospital, or another healthcare related setting. We know that a minimum of 11.7% (1176/10,007) of CO cases were hospitalized within the previous 30 days because these cases have another blood culture within this time period in our surveillance system, which only captures blood cultures from hospitalized patients. However, this underestimates prior hospitalization since we would only know of hospitalizations that included a blood culture. Moreover, we have no information about other health care exposures (e.g. dialysis or other out-patient treatments). As a more specific example of this limitation, it is likely that many of the 70 *B. pseudomallei* cases that we reported as HO, were actually community-acquired cases that were diagnosed based on blood cultures taken > 2 days after hospital admission.

Despite these limitations, this surveillance system has proved useful in characterizing the public health threat of BSI on many levels: from improving clinical care to deepening regional understanding of BSI epidemiology. By integrating BSI surveillance into routine clinical laboratory systems, our surveillance provided treating physicians and local partners with accurate, rapid pathogen identification and antimicrobial susceptibility profiles, which supported appropriate antimicrobial therapy use. Our clinical partners reiterated this message consistently and observed improvements in patient outcomes for *B. psuedomallei* cases after implementation of the automated blood culturing systems support their impressions [28, 31]. BSI surveillance has facilitated timely responses to public health threats [32, 33], enabled evaluation of new diagnostic tests [34, 35] and incidence and trends in antimicrobial resistance [36–38], and raised awareness of regional emerging infectious diseases, such as community-associated *Acinetobacter* bacteremia, *S. suis* bacteremia, and bacteremic melioidosis in a part of Thailand not traditionally considered highly endemic for that disease [28, 30, 39]. BSI surveillance has contributed to international reports and national health policy evaluations [40, 41]. We expect these data will also contribute to future evaluations of key policy issues such as consideration of PCV and the recent decision to add Hib vaccine to Thailand’s Expanded Program on Immunization and strategies to prevent further spread of anitmicrobial resistant pathogen.

Official Thai population projections indicate that between 2010 and 2030 the number of persons 50 years and older will grow by nearly 40% with the largest population growth occurring in the oldest age-groups [20, 19]. Given the high BSI incidence among persons 50 years and older, public health officials can expect corresponding increases in the BSI disease burden. In addition to the costs of acute care, public health officials can also expect increased costs for long-term care as BSI survivors suffer from cognitive impairment and functional disabilities [42].

Improvements in blood culturing systems, BSI surveillance, and other laboratory strengthening efforts are needed to promptly identify, characterize, and control the increasing threat of BSI outbreaks at their source, prevent further emergence of antimicrobial resistance, and enhance global health security. In our experience, these systems are sustainable on a local level. US CDC supported BSI surveillance stopped in Sa Kaeo province as of 2015; however, the Sa Kaeo Provincial Health Office continues automated blood culturing, together with antimicrobial susceptibility testing and infection control measures. Nakhon Phanom provincial health authorities have also assumed responsibility for supporting clinically indicated blood cultures. In addition to improved blood culturing systems, population-based BSI surveillance systems have been shown to be cost-effective in low resource settings [43] and should be expanded in other parts of SE Asia. Our finding demonstrate that BSI surveillance systems should include all health facilities performing blood cultures and incorporate laboratory strengthening activities to ensure accurate measurement of the full BSI disease burden, facilitate rapid detection and control of outbreaks at their source, and thereby enhance global health security.

## Conclusions

High CO BSI incidence in rural Thailand demonstrates the need for on-going surveillance with laboratory strengthening to improve clinical care and prevent further emergence of antimicrobial resistance.

## Additional file

Additional file 1: **Figure S1.** Community-onset (CO) BSIs caused by Gram-negative pathogens by month in Sa Kaeo and Nakhon Phanom provinces, Thailand, 2007–2014. (PDF 156 kb)

## Abbreviations

°C: Celsius
BSI: Bloodstream infection
CI: Confidence Intervals
CO: Community-Onset (CO
CRE: Carbapenem-Resistant Enterobacteriaceae
EQA: External Quality Assurance
ESBL: Extended Spectrum Beta-Lactamase
FA: FAN Aerobic (FA)
HO: Hospital-Onset (HO)
ICUs: Intensive Care Units
MB: Mycobacteria Blood (MB)
MDR: Multi-Drug Resistant
MRSA: Methicillin-Resistant S. aureus
NESDB: National Economic and Social Development Board
NIH: National Institute of Health
NP: Nakhon Phanom (NP)
PCR: PCR
PF: Pediatric FAN (PF)
PneumoADIP: Pneumococcal Vaccines Accelerated Development and Introduction Plan
SAS: Statistical Analysis Software
SK: Sa Kaeo
US CDC: United States Center for Disease Control and Prevention
