# Supplementary material for: Population-based bloodstream infection surveillance in rural Thailand, 2007–2014
Source: BMC Public Health. 2019 May 10;19(Suppl 3):521. doi: 10.1186/s12889-019-6775-4 (PMC6696817; doi:10.1186/s12889-019-6775-4)

Supplemental Figure 1. Community-onset (CO) BSIs caused by Gram-negative pathogens by month in Sa Kaeo and Nakhon Phanom provinces, Thailand, 2007-2014

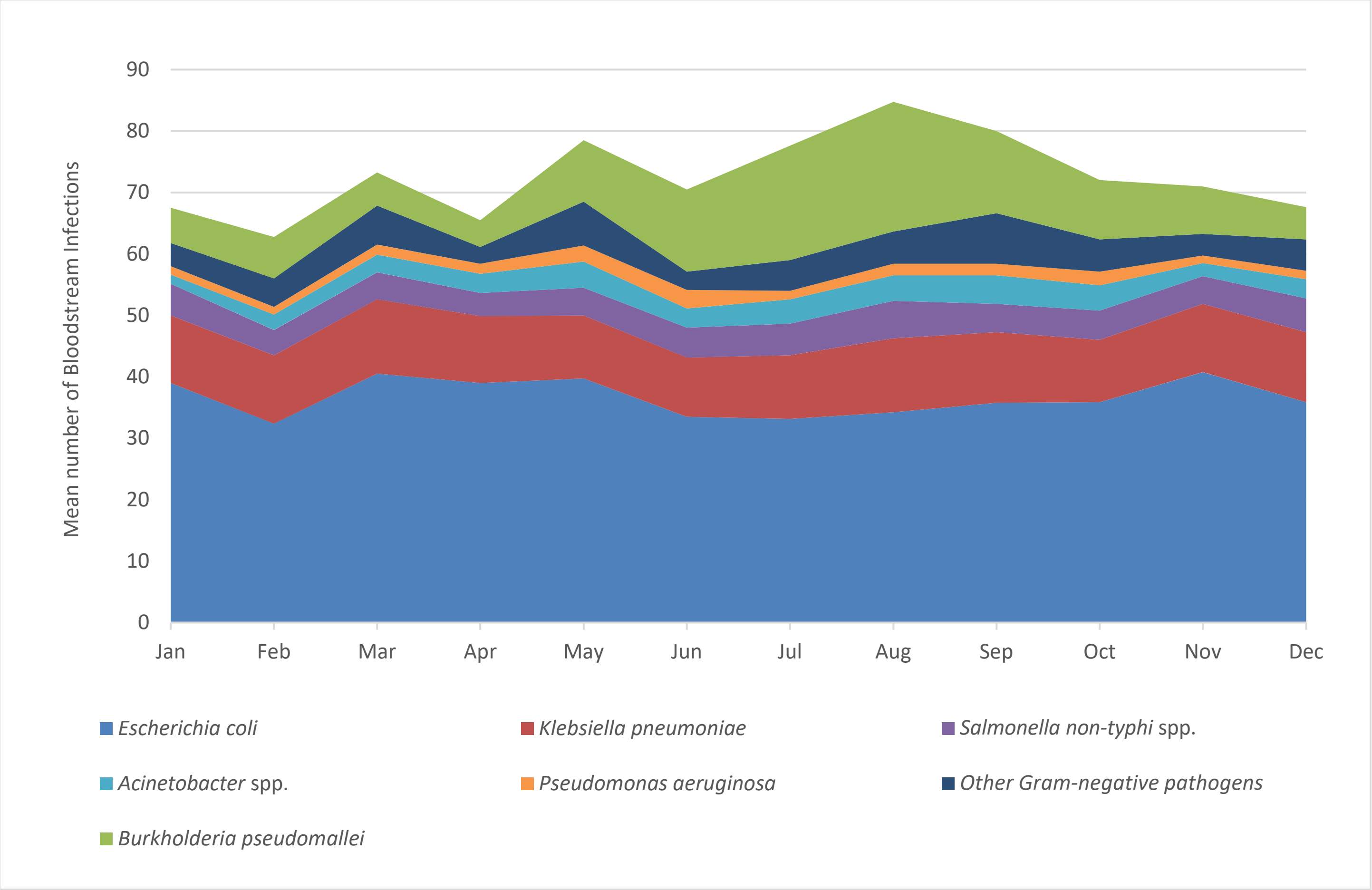

Supplement: Supplementary file 1 — Figure S1. Community-onset (CO) BSIs caused by Gram-negative pathogens by month in Sa Kaeo and Nakhon Phanom provinces, Thailand, 2007–2014. (PDF 156 kb) [file 12889_2019_6775_MOESM1_ESM.pdf]
